# Clinical risk factors for admission with *Pseudomonas* and multidrug-resistant *Pseudomonas* community-acquired pneumonia

**DOI:** 10.1186/s13756-022-01137-4

**Published:** 2022-07-14

**Authors:** Adeniyi J. Idigo, J. Michael Wells, Matthew L. Brown, Howard W. Wiener, Russell L. Griffin, Gary Cutter, Sadeep Shrestha, Rachael A. Lee

**Affiliations:** 1 Department of Epidemiology, School of Public Health, University of Alabama at Birmingham, 1665 University Boulevard, Birmingham, Alabama 35233 USA; 2 Division of Pulmonary, Allergy, and Critical Care Medicine, University of Alabama at Birmingham, Birmingham, USA; 3 University of Alabama at Birmingham Lung Health Center, Birmingham, USA; 4 Birmingham VA Medical Center, Birmingham, AL USA; 5 Department of Pharmacy, University of Alabama at Birmingham Hospital, Birmingham, USA; 6 Department of Biostatistics, School of Public Health, University of Alabama at Birmingham, Birmingham, USA; 7 Division of Infectious Diseases, University of Alabama at Birmingham School of Medicine, Birmingham, USA

**Keywords:** Community-acquired pneumonia, *Pseudomonas*, Multidrug-resistant *Pseudomonas*, Chronic obstructive pulmonary disease

## Abstract

**Background:**

Microbial etiology for community-acquired pneumonia (CAP) is evolving with pathogens known for high CAP mortality e.g., *Pseudomonas* species. Chronic obstructive pulmonary disease (COPD) patients are at risk for hospitalization for CAP. Understanding regional patterns and risk factors for multidrug-resistant (MDR) *Pseudomonas* acquisition has implications for antimicrobial stewardship.

**Objectives:**

To evaluate the regional epidemiology of MDR *Pseudomonas* CAP and its association with COPD.

**Methods:**

We queried the electronic medical records of the University of Alabama at Birmingham Healthcare System to identify patients hospitalized for CAP with *Pseudomonas* positive respiratory samples between 01/01/2013–12/31/2019. Log binomial regression models were used to examine associations between COPD diagnosis and risk of *Pseudomonas*/MDR *Pseudomonas* CAP.

**Results:**

Cohort consisted of 913 culture positive CAP cases aged 59-year (IQR:48–68), 61% (560) male, 60% (547) white, 65% (580) current/past smokers, and 42% (384) COPD. Prevalence of *Pseudomonas* CAP in culture positive CAP was 18% (167), MDR *Pseudomonas* CAP in *Pseudomonas* CAP was 22% (36), and yearly incidence of MDR *Pseudomonas* CAP was stable (*p* = 0.169). COPD was associated with *Pseudomonas* CAP (RR 1.39; 95% CI 1.01, 1.91; *p* = 0.041) but not with MDR *Pseudomonas* CAP (0.71; 95% CI 0.35, 1.45; *p* = 0.349). Stroke (RR 2.64; 95% CI 1.51, 4.61; *p* = 0.0006) and use of supplemental oxygen (RR 2.31; 95% CI 1.30, 4.12; *p* = 0.005) were associated with MDR *Pseudomonas* CAP.

**Conclusion:**

Incidence of MDR *Pseudomonas* CAP was stable over time. COPD was associated with *Pseudomonas* CAP but not with MDR *Pseudomonas* CAP. Larger cohort studies are needed to confirm findings.

## Background

Community-acquired pneumonia (CAP) is the leading infectious cause of death [1]. There are different risk factors for hospitalization for CAP, however chronic obstructive pulmonary disease (COPD) is the most common in adults [2, 3]. Annually, an estimated 5832 COPD patients per 100,000 adult population in the United States (US) are hospitalized due to CAP [3]. Additionally, chronic lower respiratory diseases, which includes COPD, was the fourth leading cause of death in the US in 2019 with 156,979 deaths (age-adjusted death rate of 38.2 per 100,000 population); in Alabama, it was the third leading cause of death with 3530 deaths (age-adjusted death rate of 55.6 per 100,000 population) [4].

Traditionally, *Streptococcus pneumoniae* (pneumococcus) is the most common bacteria isolate in CAP; other common bacterial isolates include *Hemophilus influenzae, Moraxella catarrhalis*, and atypical bacteria *(*i.e., *Mycoplasma pneumoniae, Chlamydia pneumoniae*) [5–7]. But, over the years, there has been a decline in the prevalence of pneumococcal pneumonia, especially in the US. The decline in pneumococcal pneumonia has been majorly linked to an increase in pneumococcal vaccination [8–11]. Also in the last decade, there were some reports of the emergence of CAP caused by bacteria that are conventionally not implicated in CAP including *Pseudomonas* and methicillin resistant *Staphylococcus aureus* (MRSA) [12, 13]. The proportion of CAP due to these bacteria could differ by region and time such that there is a need for local and temporal risk assessment.

Lack of identifying CAP due to *Pseudomonas* and resistant *Pseudomonas* may lead to inappropriate antimicrobial treatment, which can worsen CAP morbidity and mortality, increase the risk for antimicrobial resistance, and increase healthcare utilization and cost [14]. Furthermore, considering that pneumonia caused by *Pseudomonas* is associated with increased mortality [15, 16], understanding the regional epidemiology and antibiotic resistance profile of these bacteria is important in CAP management, especially in vulnerable population which includes those with COPD comorbidity. This study aims to assess the local epidemiology of *Pseudomonas* and multidrug-resistant *Pseudomonas*, and the association of COPD comorbidity with these bacteria in patients hospitalized with community-acquired bacterial pneumonia who had positive *Pseudomonas* isolates from respiratory tract samples.

## Methods

### Study design and population

This was a retrospective clinical cohort study of patients that were admitted to the University of Alabama at Birmingham (UAB) Healthcare System between 01/01/2013—12/31/2019 with a bacterial pneumonia diagnosis. We used bacterial pneumonia diagnosis from International Classification of Diseases (ICD) codes which include ICD 9 (481, 482, 483) and ICD 10 (J13, J14, J15, and J16) to identify patients. Only ICD 9 and 10 codes designated as ‘final’ and/or ‘confirmed’ in the electronic medical records (EMRs) were considered for disease diagnosis. Also, we used a base cohort of hospital inpatients aged 18 years or older admitted from a physician’s office or a non-healthcare facility and who had bacterial pneumonia diagnosis. A patient in the base cohort must have bacterial pneumonia diagnosis recorded in the EMRs to be present on admission. In cases where there was bacterial pneumonia diagnosis but no information about it being present on admission, the patient must have a microbiology culture sample collected within 48 h of admission. From the base cohort, we excluded those with cystic fibrosis, bronchiectasis, no respiratory samples (sputum, bronchoalveolar lavage, bronchial wash, or tracheal aspirate), no culture isolates, and those with isolates from samples collected after 48 h from admission. For patients with multiple episodes of hospitalization, only the first episode was included. The University of Alabama at Birmingham Institutional Review Board approved this study.

### Data and data source

Data was obtained from EMRs through the UAB Informatics for Integrating Biology and the Bedside (i2b2) program. The i2b2 program is an NIH-funded National Center for Biomedical Computing based at the Partners HealthCare System. We obtained data on patients’ socio-demographic characteristics; microbial culture and susceptibility; hospitalization, comorbidities, and other clinical records. We used patients’ comorbidities and validated weights to calculate Charlson comorbidity index (CCI) [17, 18].

### Outcomes

The primary outcomes were cases of CAP with 1) *Pseudomonas* and 2) multidrug-resistant (MDR) *Pseudomonas* isolates. Multidrug-resistant *Pseudomonas* isolate was defined as a *Pseudomonas* isolate that is non-susceptible (resistant or intermediate susceptibility) to at least one antipseudomonal antibiotic in three or more different antibiotics classes (carbapenems [meropenem or imipenem], cephalosporins [ceftazidime or cefepime], piperacillin/tazobactam, fluoroquinolones [ciprofloxacin or levofloxacin], aztreonam, aminoglycosides [amikacin, tobramycin, or gentamicin]) [19].

### Risk factors

The primary risk factor was a COPD diagnosis before or during hospitalization with bacterial pneumonia diagnosis. Comorbidity with COPD was identified with ICD 9 codes (490, 491, 492, 495, 496, 506, 506.4) and ICD 10 codes (J40, J41, J42, J43, J44) designated as ‘final’ and/or ‘confirmed’. We classified COPD based on time of diagnosis. Patients who had their first diagnosis of COPD during the current admission were classified as non-pre-existing COPD; those diagnosed with COPD before the current admission were classified as pre-existing COPD.

### Statistical analysis

Descriptive statistics, means, standard deviations, median, interquartile ranges and frequencies were computed and compared with chi-square tests, Fisher Exact tests, t-tests, or Mann–Whitney tests where appropriate. Log binomial regression models were used to examine associations between COPD diagnosis and risk of *Pseudomonas* and MDR *Pseudomonas* CAP. Covariates that had significant associations with the outcome, with p-value < 0.05, were included in the final (adjusted) models in addition to COPD and socio-demographic characteristics. Model Goodness of Fit was assessed and used to select the final models. The final model for the risk of *Pseudomonas* CAP included COPD, age, smoking, admission source, culture collection site, BMI, diagnosis for dependence on supplemental oxygen, and Charlson comorbidity index. The final model for the risk of MDR *Pseudomonas* CAP included COPD, age, diagnosis for dependence on supplemental oxygen, and stroke. Risk ratio (RR), 95% confidence interval, and p-value were reported. A Poisson regression model was used to estimate the annual rate ratio for the incidence of MDR *Pseudomonas* CAP; we used the natural logarithm of the annual total number of CAP cases with *Pseudomonas* isolates as offset, and we accounted for overdispersion. We used an alpha level of 0.05 for significance testing. SAS version 9.4 software (SAS Institute, Cary, NC) was used for statistical analyses.

## Results

### Isolates

**Fig. 1:**
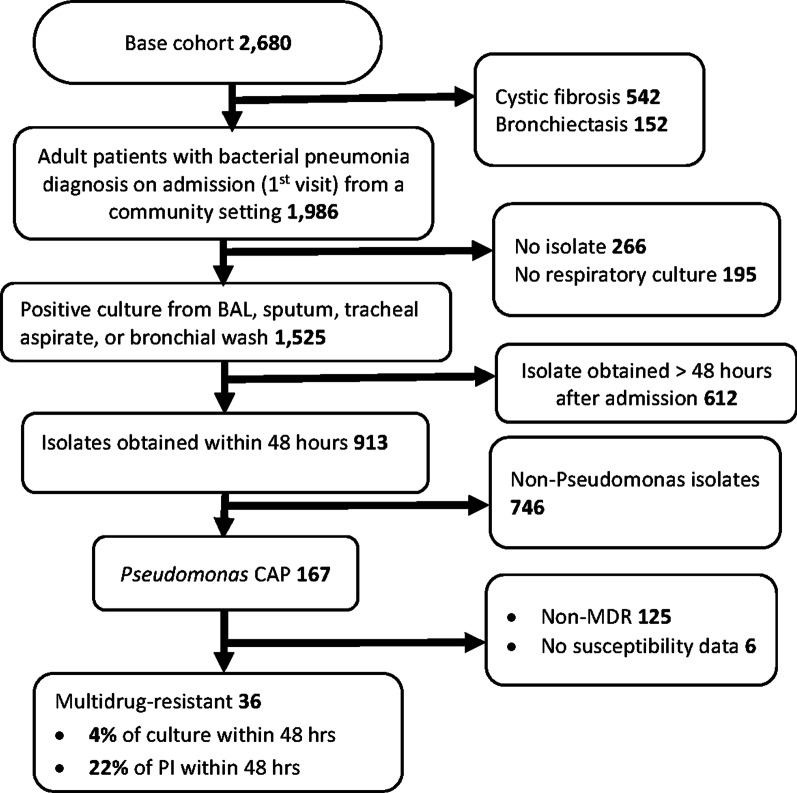
Cohort’s flowchart for *Pseudomonas* isolates. Base cohort: Hospital inpatients aged 18 years or older admitted from a physician’s office or a non-healthcare facility and who had bacterial pneumonia diagnosis. Patients must have bacterial pneumonia diagnosis recorded in the electronic medical records to be present on admission. In cases where there was bacterial pneumonia diagnosis but no information about it being present on admission, the patient must have a microbiology culture sample collected within 48 h of admission. PI: *Pseudomonas* isolate; hrs: hours; CAP: community-acquired pneumonia

A total of 1986 patients were admitted from community settings (home or physician office) with bacterial pneumonia diagnosis present on admission, or microbiology culture sample collected within 48 h of admission in cases where there was bacterial pneumonia diagnosis but no information about it being present on admission (Fig. 1). Of the 1986 patients, 77% (1525) had culture-positive respiratory samples (BAL, sputum, tracheal aspirate, or bronchial wash), meaning isolates were identified. A total of 913 patients had a culture positive respiratory sample that was collected within 48 h of admission, constituting 46% of all CAP cases (1986). Among the 913 patients, there were 167 patients with *Pseudomonas* isolates. A total of 163 (98%) of the *Pseudomonas* isolates were *Pseudomonas aeruginosa*–the remaining 4 (2%) were *Pseudomonas fluorescens*. The prevalence of *Pseudomonas* CAP was 8% of all patients admitted with CAP (1986), and 18% of those who had culture positive respiratory samples that were collected within 48 h of admission (913). Among the 167 patients with *Pseudomonas* CAP, 36 (22%) were identified as MDR. The prevalence of MDR *Pseudomonas* CAP was 2% of patients admitted with CAP (1986), and 4% of patients who had culture positive respiratory samples that were collected within 48 h of admission (913).

### Respiratory culture collected ≤ 48 h after admission

Patients had median age of 59 years (interquartile range [IQR]: 48–68), and were mostly males (61%, 560), whites (60%, 547), current/past smokers (65%, 580), and overweight or obese (54%, 491), as shown in Table 1. Also, most of the patients were admitted from home (84%, 770), and had microbiology culture obtained from sputum (39%, 353). There were 384 (42%) patients diagnosed with COPD and the median Charlson comorbidity index for all patients was 4 (IQR: 2–7). In unadjusted analysis, COPD was associated with *Pseudomonas* isolation when compared to those with no COPD diagnosis (23.8% vs 14.3%; *p* = 0.0002). The association was more evident when those with pre-existing COPD were compared to those with COPD diagnosis during CAP admission and those with no COPD diagnosis (28.1% vs 11.3% vs 14.3%; p < 0.0001). Other characteristics associated with *Pseudomonas* isolation in unadjusted analyses included underweight/normal BMI (BMI < 25 vs ≥ 25: 22.9% vs 14.3%; *p* = 0.0008), culture collection site (bronchial wash 30.0%, sputum 21.8%, tracheal aspirate 18.2%, BAL 10.1%; *p* = 0.0013), Charlson comorbidity index (*p* = 0.011), medical intensive care unit admission (*p* = 0.0004), diagnosis for dependence on supplemental oxygen (p < 0.0001).

**Table 1:** Comparison of patients with *Pseudomonas* isolates with those without *Pseudomonas* isolates in a cohort of patients with community-acquired bacterial pneumonia (N = 913)

Characteristics	N (%)	Culture ( +) for *Pseudomonas* N = 167 (18.3%)	Culture ( +) not for *Pseudomonas* N = 746 (81.7%)	*p* value
Age in years, median	59 (48–68)	60 (50–70)	59 (48–68)	0.301
Age				0.206
< 65 years	612 (67.0)	105 (62.9)	507 (68.0)	
$\ge$ 65 years	301 (33.0)	62 (37.1)	239 (32.0)	
Sex				0.248
Male	560 (61.3)	109 (65.3)	451 (60.5)	
Female	353 (38.7)	58 (34.7)	295 (39.5)	
Race				0.901
Black	335 (36.7)	59 (35.3)	276 (37.1)	
White	547 (60.0)	102 (61.1)	445 (59.7)	
Others	30 (3.3)	6 (3.6)	24 (3.2)	
Smoking				0.420
Current/past smoker	580 (65.0)	110 (65.9)	470 (64.8)	
Never smoker	281 (31.5)	54 (32.3)	227 (31.3)	
Unknown	31 (3.5)	3 (1.8)	28 (3.9)	
Body mass index (Kg/m^2^)				**0.005**
< 18.5	97 (10.7)	25 (15.2)	72 (9.7)	
18.5–24.9	318 (35.1)	70 (42.4)	248 (33.5)	
25.0–29.9	217 (24.0)	35 (21.2)	182 (24.6)	
≥ 30.0	274 (30.2)	35 (21.2)	239 (32.3)	
Culture collection site				**0.001**
Sputum	353 (38.7)	77 (46.1)	276 (37.0)	
Bronchoalveolar lavage	207 (22.7)	21 (12.6)	186 (24.9)	
Bronchial wash	40 (4.4)	12 (7.2)	28 (3.8)	
Tracheal aspirate	313 (34.3)	57 (34.1)	256 (34.3)	
Admission source				0.065
Home	770 (84.3)	133 (79.6)	637 (85.4)	
Physician office	143 (15.7)	34 (20.4)	109 (14.6)	
Health Insurance				0.131
Medicaid	146 (16.0)	30 (18.0)	116 (15.6)	
Medicare	414 (45.4)	84 (50.3)	330 (44.2)	
Financial assistance	24 (2.6)	3 (1.8)	21 (2.8)	
Private	242 (26.5)	42 (25.2)	200 (26.8)	
Others	87 (9.5)	8 (4.8)	79 (10.6)	
COPD diagnosis, based on time of pneumonia admission				** < 0.0001**
Pre-existing COPD	278 (30.6)	78 (47.3)	200 (26.9)	
Non-pre-existing COPD	106 (11.7)	12 (7.3)	94 (12.6)	
No COPD diagnosis	526 (57.8)	75 (45.5)	451 (60.5)	
Asthma				0.201
Yes	120 (13.1)	27 (16.2)	93 (12.5)	
No	793 (86.9)	140 (83.8)	653 (87.5)	
HF				0.477
Yes	289 (31.7)	49 (29.3)	240 (32.2)	
No	624 (68.4)	118 (70.7)	506 (67.8)	
Stroke				0.911
Yes	73 (8.0)	13 (7.8)	60 (8.0)	
No	840 (92.0)	154 (92.2)	686 (92.0)	
Type 2 diabetes mellitus				0.388
Yes	316 (34.6)	53 (31.7)	263 (35.3)	
No	597 (65.4)	114 (68.3)	484 (64.8)	
Charlson comorbidity index, median (IQR)	4 (2–7)	4 (2—8)	4 (2—7)	**0.011**
Charlson comorbidity index				0.108
0–3	434 (47.5)	70 (41.9)	364 (48.8)	
$\ge$ 4	479 (52.5)	97 (58.1)	382 (51.2)	
MICU admission				**0.0004**
Yes	292 (32.0)	34 (20.4)	258 (34.6)	
No	621 (68.0)	133 (79.6)	488 (65.4)	
Dependence on supplemental oxygen diagnosis				** < 0.0001**
Yes	171 (18.7)	52 (31.1)	119 (16.0)	
No	742 (81.3)	115 (68.9)	627 (84.1)	
In-hospital steroid administration				0.234
Yes	443 (48.5)	88 (52.7)	355 (47.6)	
No	470 (51.5)	79 (47.3)	391 (52.4)	
Length of hospital stay (days), median (IQR)	10 (5–19)~	9 (5–16)	10 (6–19)	0.105
In-hospital death				0.685
Yes	133 (14.6)	26 (15.6)	107 (14.3)	
No	780 (85.4)	141 (84.4)	639 (85.7)	

Median (interquartile range) reported for age, Charlson comorbidity index, length of hospital stay, and N (%) reported for others; % may not add up to 100% due to approximation

*p*-values in bold are < 0.05

In adjusted models (Table 2), patients with pre-existing COPD had 39% higher risk of *Pseudomonas* isolation than those with no COPD diagnosis (RR 1.39; 95% CI 1.01, 1.91; *p* = 0.041). Also, patients who had diagnosis for dependence on supplemental oxygen (RR 1.58; 95% CI 1.16, 2.15; *p* = 0.004), and those with underweight or normal BMI had higher risk of *Pseudomonas* isolation (RR 1.70; 95% CI 1.20, 2.41; *p* = 0.003).

**Table 2:** COPD as a risk factor for *Pseudomonas* isolation among hospitalized patients with community-acquired pneumonia

	RR (95% CI )	*p *value
COPD diagnosis		
Pre-existing COPD	1.39 (1.01, 1.91)	**0.041**
No COPD diagnosis	Ref	Ref
Dependence on supplemental oxygen		
Yes	1.58 (1.16, 2.15)	**0.004**
No	Ref	Ref
BMI		
< 25	1.70 (1.20, 2.41)	**0.003**
25.0–29.9	1.32 (0.86, 2.01)	0.202
≥ 30.0	Ref	Ref
MICU		
Yes	0.63 (0.44, 0.91)	**0.013**
No	Ref	Ref

Model adjusted for chronic obstructive pulmonary disease (COPD), BMI, dependence on supplemental oxygen diagnosis, age, smoking, admission source, culture collection source, and Charlson comorbidity index

*p*-values in bold are < 0.05

Among patients with *Pseudomonas* isolates, there was no yearly trend in the admission of patients with MDR *Pseudomonas* isolates (RR 1.05; 95% CI 0.98, 1.14; *p* = 0.169). Also, COPD was not associated with MDR *Pseudomonas* isolates (pre-existing COPD RR 0.71; 95% CI 0.35, 1.45; *p* = 0.349), Tables 3 & 4. However, stroke (RR 2.64; 95% CI 1.51, 4.61; *p* = 0.0006), diagnosis for dependence on supplemental oxygen (RR 2.31; 95% CI 1.30, 4.12; *p* = 0.005), and 10-year increase in age (RR 0.83; 95% CI 0.69, 0.99; *p* = 0.043) were associated with MDR *Pseudomonas* isolates. There were no statistically significant associations between COPD and individual antibiotic classes and individual antibiotics in each class.

**Table 3:** Pattern of multidrug-resistant *Pseudomonas* isolates in *Pseudomonas* positive respiratory culture collected within 48 h of admission (N = 161)

Characteristics	N (%)	+ MDR N (%) 36 (22.4%)	-MDR N (%) 125 (77.6%)	p-value
**Age** in years, median	60 (51–70)	57 (43–69)	62 (53–71)	**0.035**
Age				0.303
< 65 years	100 (62.1)	25 (69.4)	75 (60.0)	
$\ge$ 65 years	61 (37.9)	11 (30.6)	50 (40.0)	
Sex				0.849
Male	105 (65.2)	23 (63.9)	82 (65.6)	
Female	56 (34.8)	13 (36.1)	43 (34.4)	
Race				0.830
Black	59 (36.7)	15 (41.7)	44 (35.2)	
White	96 (59.3)	20 (55.6)	76 (60.8)	
Others	6 (3.7)	1 (2.8)	5 (4.0)	
Smoking				**0.034**
Current/past smoker	107 (66.5)	18 (50.0)	89 (71.2)	
Never smoker	51 (31.7)	17 (47.2)	34 (27.2)	
Unknown	3 (1.9)	1 (2.8)	2 (1.6)	
Body mass index (Kg/m^2^)				0.144
< 18.5	24 (15.1)	8 (22.9)	16 (12.9)	
18.5–24.9	67 (42.1)	16 (45.7)	51 (41.1)	
25.0–29.9	34 (21.4)	3 (8.6)	31 (25.0)	
≥ 30.0	34 (21.4)	8 (22.9)	26 (21.0)	
Culture collection site				**0.047**
Sputum	73 (45.3)	12 (33.3)	61 (48.8)	
Bronchoalveolar lavage	20 (12.4)	3 (8.3)	17 (13.6)	
Bronchial wash	11 (6.8)	1 (2.8)	10 (8.0)	
Tracheal aspirate	57 (35.4)	20 (55.6)	37 (29.6)	
Admission source				0.321
Home	130 (80.8)	27 (75.0)	103 (82.4)	
Physician office	31 (19.3)	9 (25.0)	22 (17.6)	
COPD				0.248
Pre-existing COPD	75 (47.2)	15 (41.7)	60 (48.8)	
Non-pre-existing COPD	12 (7.6)	1 (2.8)	11 (8.9)	
No COPD diagnosis	72 (45.3)	20 (55.6)	52 (42.3)	
Asthma				0.985
Yes	27 (16.8)	6 (16.7)	21 (16.8)	
No	134 (83.2)	30 (83.3)	104 (83.2)	
HF				0.986
Yes	49 (30.4)	11 (30.6)	38 (30.4)	
No	112 (69.6)	25 (69.4)	87 (69.6)	
Stroke				**0.010**
Yes	13 (8.1)	7 (19.4)	6 (3.7)	
No	148 (91.9)	29 (80.6)	119 (95.2)	
Type 2 diabetes mellitus				0.870
Yes	51 (31.7)	11 (30.6)	40 (32.0)	
No	110 (68.3)	25 (69.4)	85 (68.0)	
Charlson comorbidity index, median (IQR)	4 (3—8)	4 (2—6)	5 (3—9)	0.217
Charlson comorbidity index				0.633
0–3	66 (41.0)	16 (44.4)	50 (40.0)	
$\ge$ 4	95 (59.0)	20 (55.6)	75 (60.0)	
MICU admission				0.228
Yes	34 (21.1)	5 (13.9)	29 (23.2)	
No	127 (78.9)	31 (86.1)	96 (76.8)	
Dependence on supplemental oxygen diagnosis				**0.030**
Yes	52 (32.3)	17 (47.2)	35 (28.0)	
No	109 (67.7)	19 (52.8)	90 (72.0)	
In-hospital steroid administration				0.178
Yes	83 (51.6)	15 (41.7)	68 (54.4)	
No	78 (48.5)	21 (58.3)	57 (45.6)	
Length of hospital stay (days), median (IQR)	9 (5–16)	8 (5–16)	9 (5–16)	0.564
In-hospital Death		8 (5–16)	9 (5–16)	0.148
Yes	26 (16.2)	3 (8.3)	23 (18.4)	
No	135 (83.9)	33 (91.7)	102 (81.6)	

Antipseudomonal antibiotic classes with specific antibiotics: Fluoroquinolones (ciprofloxacin or levofloxacin); 3^rd^/4^th^-geberation cephalosporins (ceftazidime or cefepime); aminoglycosides (tobramycin or amikacin or gentamicin); carbapenem (imipenem or meropenem)

Multidrug-resistance: non-susceptibility (resistance or intermediate susceptibility) to at least one antibiotic in three or more antipseudomonal antibiotics classes above

MDR: multidrug-resistant

Median (interquartile range) reported for age, Charlson comorbidity index, length of hospital stay, and N (%) reported for others; % may not add up to 100% due to approximation﻿

*p*-values in bold are < 0.05

Out of the 167 patients who had culture-positive *Pseudomonas* isolates, 6 did not have antibiotics susceptibility data, 36 were MDR, and 125 were non-MDR

**Table 4:** Risk ratio of multidrug-resistant *Pseudomonas* isolates (N = 161)

	RR (95% CI)	*p *value
COPD diagnosis		
Pre-existing COPD	0.71 (0.35, 1.45)	0.349
No COPD diagnosis	Ref	Ref
Stroke		
Yes	2.64 (1.51, 4.61)	**0.0006**
No	Ref	Ref
Dependence on supplemental oxygen		
Yes	2.31 (1.30, 4.12)	**0.005**
No	Ref	Ref
10-year increase in age	0.83 (0.69, 0.99)	**0.043**

Antipseudomonal antibiotic classes with specific antibiotics: Fluoroquinolones (ciprofloxacin or levofloxacin); 3^rd^/4^th^-generation cephalosporins (ceftazidime or cefepime); aminoglycosides (tobramycin or amikacin or gentamicin); carbapenem (imipenem or meropenem)

Multidrug-resistance: non-susceptibility (resistance or intermediate susceptibility) to at least one antibiotic in three or more antipseudomonal antibiotics classes above

CAP: community-acquired bacterial pneumonia, RR: risk ratio

*p*-values in bold are < 0.05

Model adjusted for age, diagnosis for dependence on supplemental oxygen diagnosis, stroke out of the 167 patients who had culture-positive *Pseudomonas* isolates, 6 did not have antibiotics susceptibility data, 36 were MDR, and 125 were non-MDR

## Discussion

In a clinical cohort of patients hospitalized with bacterial CAP between 2013 and 2019 at a tertiary hospital in the southeastern USA, we examined the epidemiology of CAP with *Pseudomonas* and MDR *Pseudomonas* isolates, and the association of this CAP with COPD comorbidity. In patients with culture-positive respiratory samples, the estimated prevalence of CAP with *Pseudomonas* isolates was 18%; among CAP patients with *Pseudomonas* isolates, the estimated prevalence of CAP with MDR *Pseudomonas* was 22%. There was no significant trend in the yearly incidence of CAP with MDR *Pseudomonas* isolates over the years. Lastly, though COPD was associated with the risk of isolating *Pseudomonas* isolates, it was not associated with the risk of isolating MDR *Pseudomonas* isolates.

There are variations in the estimated prevalence of CAP due to *Pseudomonas* and MDR *Pseudomonas* in different studies. This difference is possibly due to different populations [13, 20–24]. In an observational study by Cilloniz et al. in an European population, the estimated 15-year prevalence of CAP due to *Pseudomonas* isolates was 4% among patients with culture positive CAP [16]. This was lower than the 7-year estimated prevalence of 18% in the current study. However, the estimated prevalence of MDR *Pseudomonas* aeruginosa among *Pseudomonas aeruginosa* isolates reported by Cilloniz et al. was higher than the current study (32% vs 22%) [23]. In a multinational study by Restrepo et al., the estimated global prevalence of CAP due to *Pseudomonas aeruginosa* was 11.3% in isolate-positive CAP cases. Again, this was lower than the estimate (18.3%) for the current study which drew its population from the southeastern US. The estimated global prevalence for MDR *Pseudomonas aeruginosa*, irrespective of positive culture isolates, reported in the multinational study was 2.8%. This, again, was lower than the estimated prevalence reported in the current study (3.9%) [24]. Though the current study focused on any *Pseudomonas* isolates while the multinational study focused on *Pseudomonas aeruginosa* isolates*,* 98% of *Pseudomonas* isolates in the current study were *Pseudomonas aeruginosa*. There may be more cases of *Pseudomonas* CAP in southeastern United States when compared to the global average. Understanding validated regional differences in *Pseudomonas* CAP could help guide the clinical treatment of CAP at the regional level. It will be informative to explore region-specific characteristics that drive regional differences in the prevalence of *Pseudomonas* and MDR *Pseudomonas* CAP in future research; specifically, how regional differences in vaccination (e.g., pneumococcal vaccines), antimicrobial prescribing practices, and other factors affect regional differences in MDR *Pseudomonas* CAP epidemiology. According to the Centers for Disease Control and Prevention 2020 data on outpatient prescription of fluoroquinolones dispensed in US pharmacies, Southern US (e.g., Alabama, Mississippi) accounts for the highest prescription rates [25]. Alabama had the second highest rate of outpatient prescriptions of fluoroquinolones with 77 prescriptions per 1000 population, after Mississippi (1^st^) with 82 prescriptions per 1000 population. A lot of effort is geared toward improving rational use of antibiotics through antimicrobial stewardship [26].

Cilloniz et al., like the current study, found that COPD was associated with the risk of CAP due to *Pseudomonas*, and it was not associated with the risk of MDR *Pseudomonas *[16]. In a meta-analysis that examined risk factors for MDR *Pseudomonas aeruginosa*, previous antibiotics use and hospital admission, including intensive-care unit, were the risk factors identified [27]. It is important to note that most of the studies included in the meta-analysis had base cohorts that were not CAP. In patients with intensive care unit-acquired pneumonia due to *Pseudomonas aeruginosa*, Barat et al. found no association between COPD and risk of MDR *Pseudomonas* in adjusted models [28]. Restrepo et al., also found that chronic lung disease was associated with *Pseudomonas* CAP and MDR *Pseudomonas* CAP in their multi-nation study [24]. Different results observed by various studies with the association between COPD and MDR *Pseudomonas* suggest that a regional approach to the assessment of MDR *Pseudomonas* may be the effective means to manage this public health burden.

Diagnosis for dependence on supplemental oxygen during the current or a previous hospital visit was one of the risk factors for MDR *Pseudomonas* identified in the present study in an adjusted model. Supplemental oxygen is a medical device that provides oxygen supply to patients with low oxygen levels. Regular use of respiratory devices like supplemental oxygen could promote the formation of bacterial biofilm which could predispose patients to infections when there is poor hygiene in the handling of the device [29, 30]. This may be a potential explanation for the association between dependence on supplemental oxygen and the risk of MDR *Pseudomonas*. Also, a patient who depends on supplemental oxygen could have had a previous hospitalization which required mechanical ventilation. This is also a potential source of acquisition of MDR *Pseudomonas *[28, 31–33]. There are different indications for the use of supplemental oxygen, and COPD is one of them, especially severe one. Though we did not find any association between COPD and risk of MDR *Pseudomonas*, future research could examine the interaction between COPD and the use of supplemental oxygen and the risk of MDR *Pseudomonas*.

We found that among CAP patients with *Pseudomonas* isolates, those with current or previous stroke diagnosis were more likely to have MDR *Pseudomonas* isolates in their respiratory samples when compared to those with no stroke diagnosis. Stroke clinical management may involve frequent hospital admissions and prolong length of hospital stay [34–36]. Such frequent and prolonged patient interactions with healthcare facilities have been shown to be risk factors for acquisition of drug-resistant bacteria like *Pseudomonas *[27]. This may be the mechanism which makes stroke comorbidity to be associated with the risk of MDR *Pseudomonas* CAP. We also found that increasing age was associated with lower risk of MDR *Pseudomonas* CAP. It is possible that in this study setting known risk factors for MDR bacteria, like exposure to broad spectrum antibiotics, are lower in older patients.

While the current study had a substantial population to estimate the prevalence, incidence, and risk of *Pseudomonas* CAP, we had little sample size (161) for the analysis of the risk of MDR *Pseudomonas* among CAP patients with *Pseudomonas* isolates. We also relied on EMR for patient information, so the data we used for analysis was limited to what was obtainable in the EMR. Though EMR data might have some weaknesses, it is inexpensive and provides real-world information.

## Conclusions

In summary, we found that the incidence of MDR *Pseudomonas* CAP was stable over time, and prevalences of *Pseudomonas* and MDR *Pseudomonas* community-acquired pneumonia were different in this study population when compared to other regions, highlighting the importance of leveraging local epidemiology and validated risk factors for antimicrobial stewardship guidance. Chronic obstructively pulmonary disease was associated with *Pseudomonas* CAP but not with MDR *Pseudomonas* CAP. Larger cohort studies are needed to confirm these findings.

## Data Availability

The datasets used during the current study are available from the corresponding author on reasonable request.
